# Fabrication of parabolic Si nanostructures by nanosphere lithography and its application for solar cells

**DOI:** 10.1038/s41598-017-07463-7

**Published:** 2017-08-04

**Authors:** See-Eun Cheon, Hyeon-seung Lee, Jihye Choi, Ah Reum Jeong, Taek Sung Lee, Doo Seok Jeong, Kyeong-Seok Lee, Wook-Seong Lee, Won Mok Kim, Heon Lee, Inho Kim

**Affiliations:** 1 Electronic Materials Research Center, Korea Institute of Science and Technology, Seoul, 02792 Republic of Korea; 2 Department of Materials Science and Engineering, Korea University, Seoul, 02841 Republic of Korea; 3 Department of Materials Science and Engineering, Yonsei University, Seoul, 03722 Republic of Korea; 4 Division of Nano & Information Technology, Korea University of Science and Technology, Seoul, 02792 Republic of Korea

## Abstract

We demonstrated fabrication of a parabola shaped Si nanostructures of various periods by combined approach of nanosphere lithography and a single step CF_4_/O_2_ reactive ion etch (RIE) process. Silica nanosphere monolayers in a hexagonal array were well deposited by a solvent controlled spin coating technique based on binary organic solvents. We showed numerically that a parabolic Si nanostructure of an optimal period among various-shaped nanostructures overcoated with a dielectric layer of a 70 nm thickness provide the most effective antireflection. As the simulation results as a design guide, we fabricated the parabolic Si nanostructures of a 520 nm period and a 300 nm height exhibiting the lowest weighted reflectance of 2.75%. With incorporation of such parabolic Si nanostructures, a damage removal process for 20 sec and SiN_x_ antireflection coating of a 70 nm thickness, the efficiency of solar cells increased to 17.2% while that of the planar cells without the nanostructures exhibited 16.2%. The efficiency enhancement of the cell with the Si nanostructures was attributed to the improved photocurrents arising from the broad spectral antireflection which was confirmed by the external quantum efficiency (EQE) measurements.

## Introduction

Broadband antireflection in crystalline silicon solar cells is one of key factors to high efficiency^1, 2^. In order to suppress light reflectance from Si wafers, surface texturing of the Si wafers are adopted to refract incident light into the wafers. One of conventional texturing schemes is introduction of Si micro pyramids in the height range of 5 to 20 μm. The Si micro pyramids can be fabricated by simply dipping mono-crystalline Si wafers of (100) crystal orientation into aqueous alkaline solutions such as NaOH and KOH^3, 4^. This texturing technique is facile and cost-effective; thus, most of the solar industries have adopted it for effective light trapping in solar cells^5, 6^. However, this texturing scheme wastes Si materials of a nearly ten micrometer thickness in order to obtain a full coverage of micro pyramids on the wafers and inevitably leads to wide size distributions of micro pyramids, which causes a difficulty in the elaborate control of geometrical dimensions of pyramids. Furthermore, the height of Si pyramids in micro scale poses a challenge for its application in ultra-thin Si wafers below a thickness of 50 μm^7^. In this regard, a plethora of shallow texturing schemes with Si nanostructures in sub-micron scale have been proposed. A variety of nano lithography techniques have been demonstrated for fabrication of Si nanostructures by combining of etch masks in nano scale features with wet or dry etching^8–11^. Nanosphere lithography using self-assembled silica beads is one of cost-effective approaches to have nano scale etch masks^12–14^. The spin coating technique for deposition of self-assembled silica bead monolayers has a high potential because it can be a high throughput process compared with a conventional Langmuir-Blodgett method and nano imprinting^15–17^. The self-assembled silica beads combined with well-controlled reactive ion etch (RIE) processes can provide a versatile texturing scheme to fabricate various Si nanostructures. The fabrication of various-shaped Si nanostructures such as cone, hole, and dome by nanosphere lithography was reported to feasible and the high optical performances were demonstrated^2, 18–21^. However, the high efficiency of Si solar cells with the nanostructures of high aspect ratios was rarely demonstrated mostly because of deteriorated charge collection efficiencies caused by enlarged surface areas and excessive emitter doping^22, 23^. The Si nanostructures of a low aspect ratio combined with shallow emitter doping would be key to realization of high efficiency solar cells. In this study, we demonstrate the fabrication of parabolic Si nanostructures with deposition of silica bead monolayers followed by a single step RIE process for broad band antireflection in crystalline solar cells. The geometrical aspects such as the size, periodicity and shape of the Si nanostructures are crucial to effective antireflection^24, 25^. We adjusted the periodicity of the Si nanostructures simply by using different sizes of the silica beads. The shape and height of the Si nanostructures were tailored for effective antireflection in broad spectral ranges by optimal choices of the RIE gases and compositions^20, 26, 27^. The parabolic Si nanostructures on the Si wafers enabled strong antireflection in broad spectral ranges from 350 nm to 1100 nm owing to combinations of the effects of graded index and diffraction from nano gratings. With employing the parabolic Si nanostructures of a 520 nm period and a 300 nm height on the Si wafers, the photocurrent on average was increased by over ~2 mA/cm^2^ with reference to the solar cells without nano texturing. The parabolic Si nanostructures we demonstrate in this study would be very promising light trapping structures especially for the ultrathin wafer based Si solar cells because of the shallow texturing in submicron scale.

## Results and Discussion

### Fabrication of parabolic Si nanostructures

**Figure 1:**
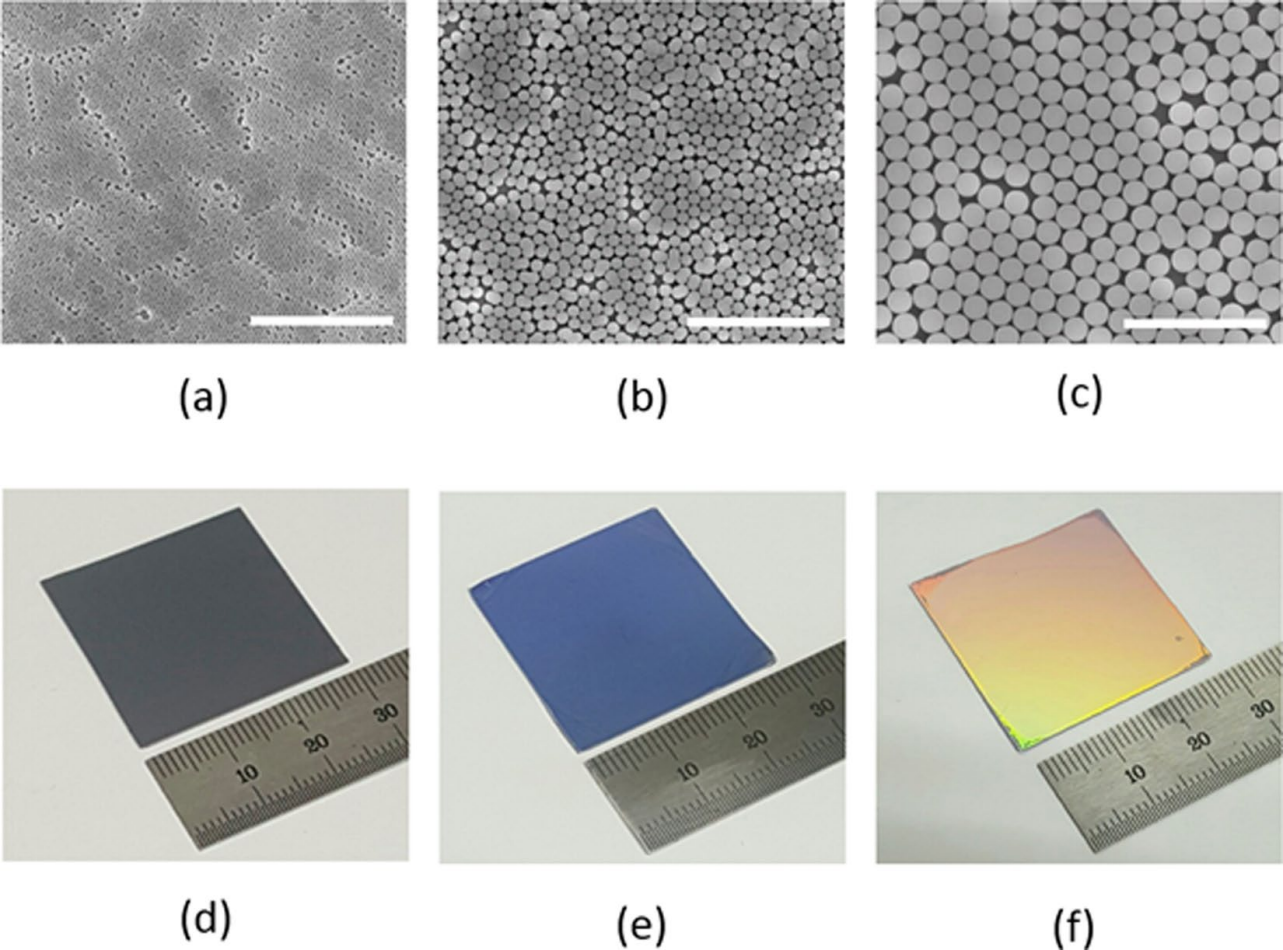
SEM images of silica bead self-assembled monolayers with different bead sizes of (**a**) 310 nm (P310), (**b**) 520 nm (P520), and (**c**) 960 nm (P960). Photo images the silica bead monolayers of (**d**) P310, (**e**) P520, and (**f**) P960 deposited on Si substrate of 3 cm × 3 cm. The scale bar in the SEM images is 5 μm.

**Figure 2:**
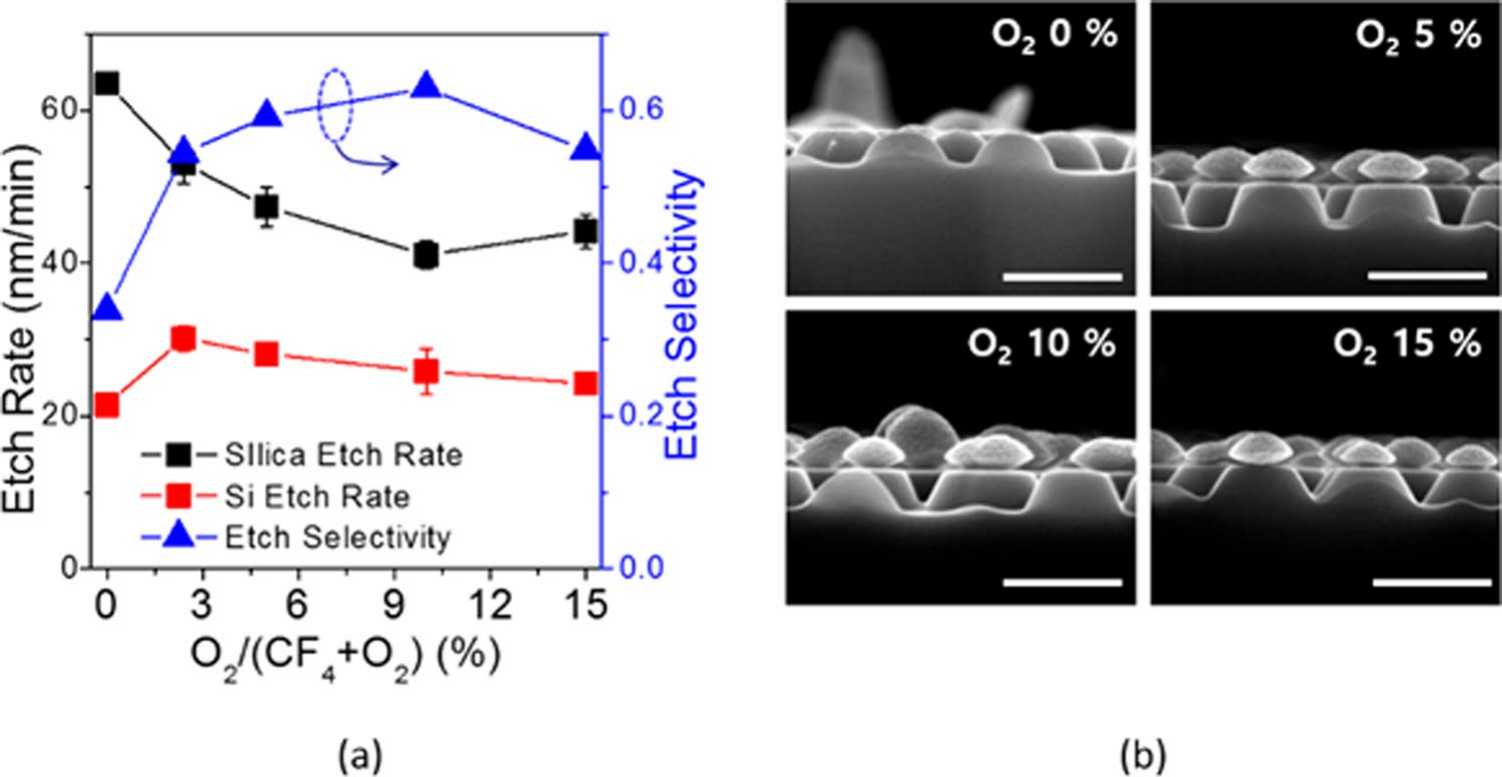
(**a**) Etch rate and etch selectivity of silica beads and Si wafers as a function of oxygen ratio in a CF_4_/O_2_ RIE process. (**b**) Cross sectional SEM images of the Si nanostructures fabricated with P520 silica beads as a mask. The oxygen ratio was varied from 0% to 15% to adjust the etch selectivity. The scale bar is 500 nm for all the SEM images.

**Figure 3:**
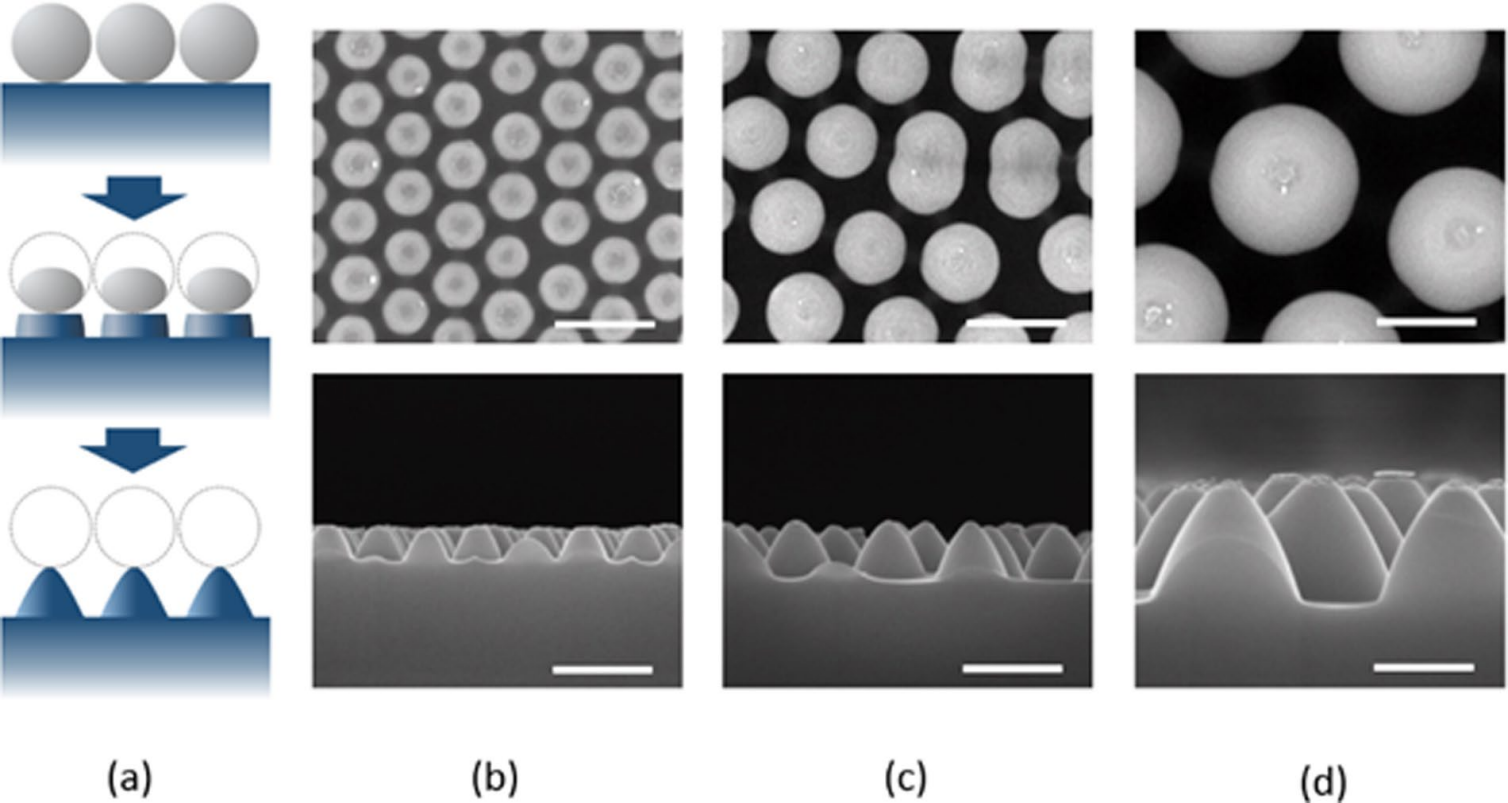
(**a**) Schematic of Si nanostructure fabrication processes with silica beads and a CF_4_/O_2_ RIE process. SEM images of the Si nanostructures after an RIE process with different sizes of the silica beads as a mask: (**b**) P360, (**c**) P520, (**d**) P960. The scale bar is 500 nm for all the SEM images.

**Figure 4:**
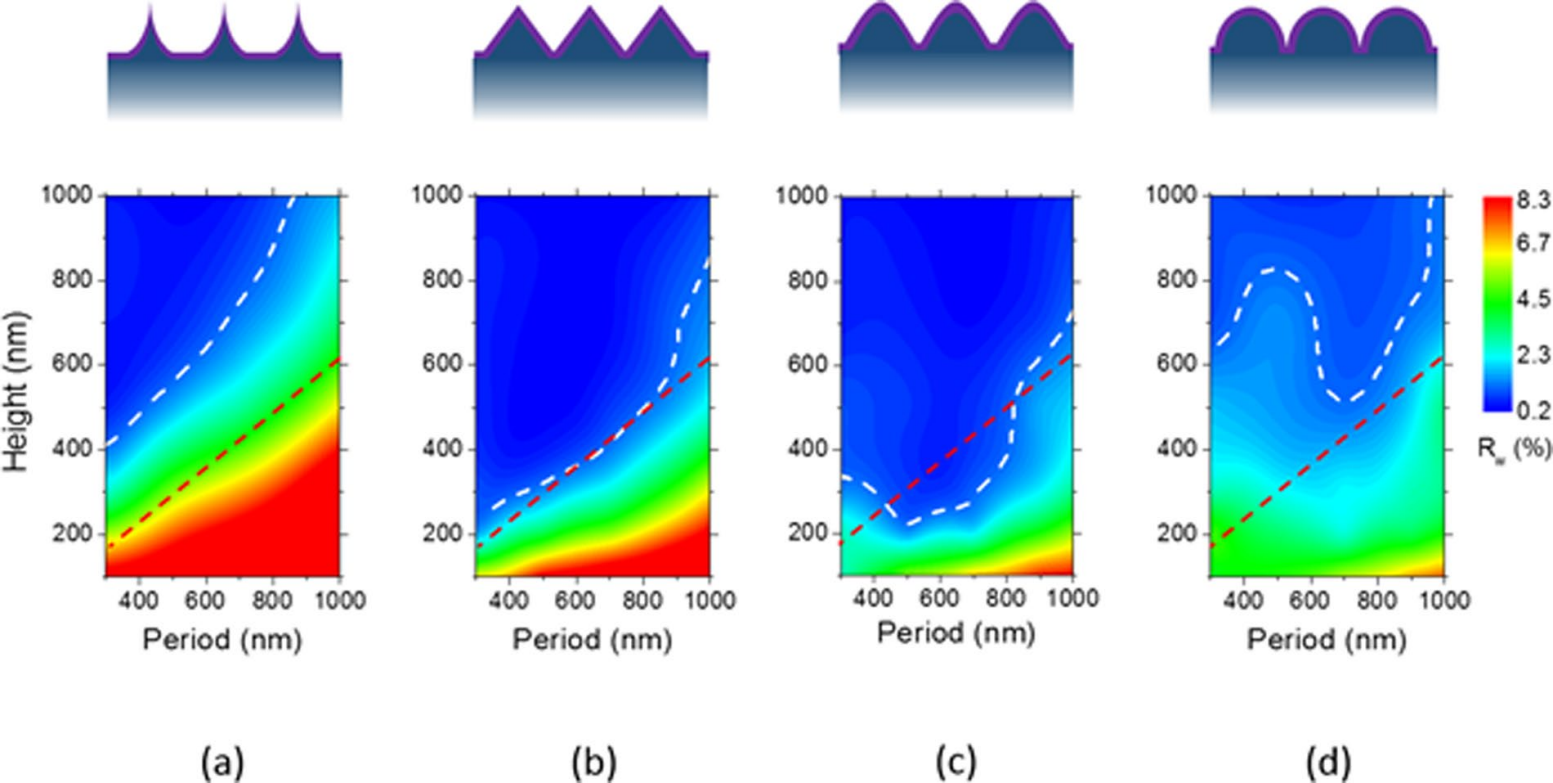
Simulated average weighted reflectance values of Si wafers with different shapes of Si nanostructures as a function of heights and periods: (**a**) curved cone, (**b**) nanocone, (**c**) paraboloid, (**e**) ellipsoid. The white dashed lines denote a contour line of a 1% reflectance. Schematics of cross sectional views of each nanostructure is illustrated together. The red dashed lines indicate the maximum height of the Si nanostructures to be achieved with a etch selectivity in the CF_4_/O_2_ RIE process in this study.

**Figure 5:**
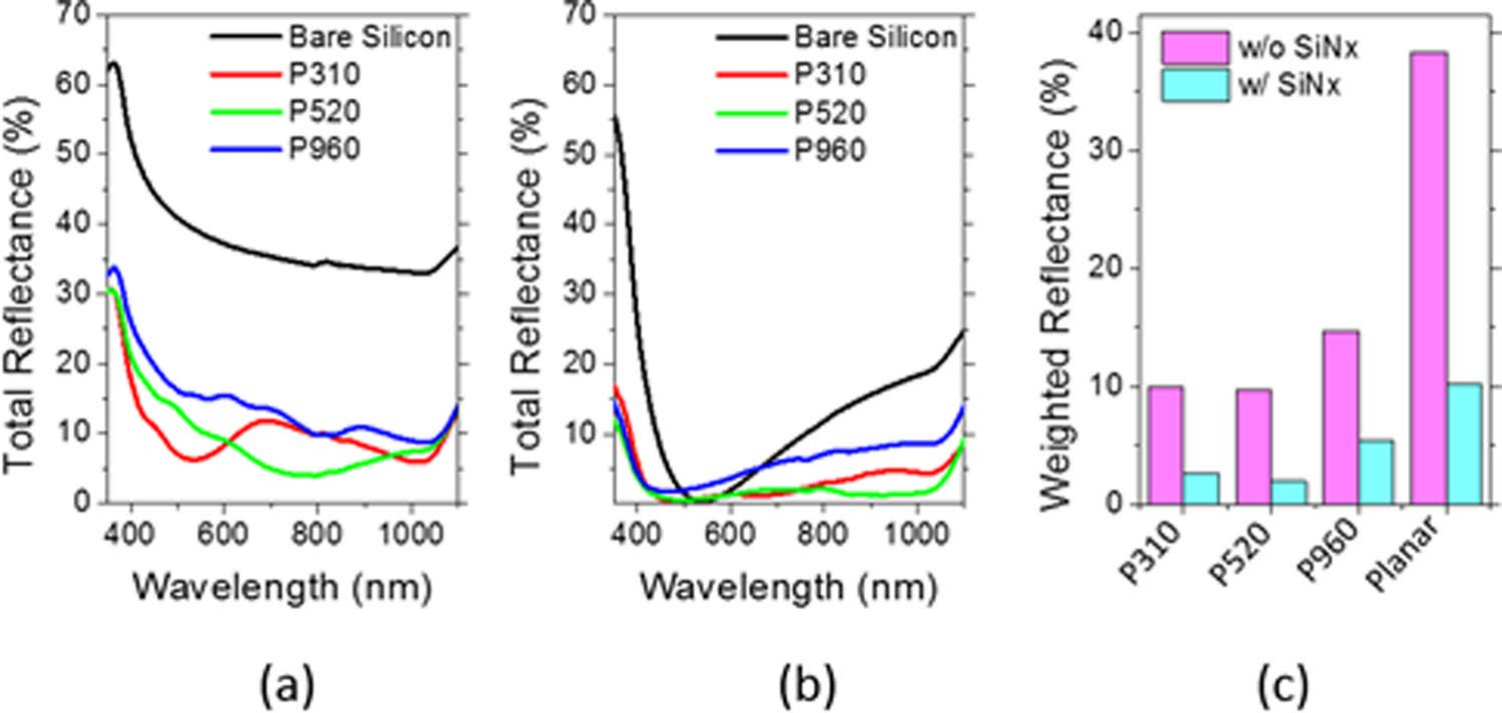
Reflectance curves of Si wafers with parabolic Si nanostructures (**a**) before and (**b**) after SiN_x_ deposition. Three different sizes of silica beads (P310, P520, P960) were used for RIE masks. Average reflectance values of the Si wafers with parabolic Si nanostructures before and after SiN_x_ deposition. Reflectance values of planar Si wafers (bare Si) without nanostructures are also shown together for comparison.

**Figure 6:**
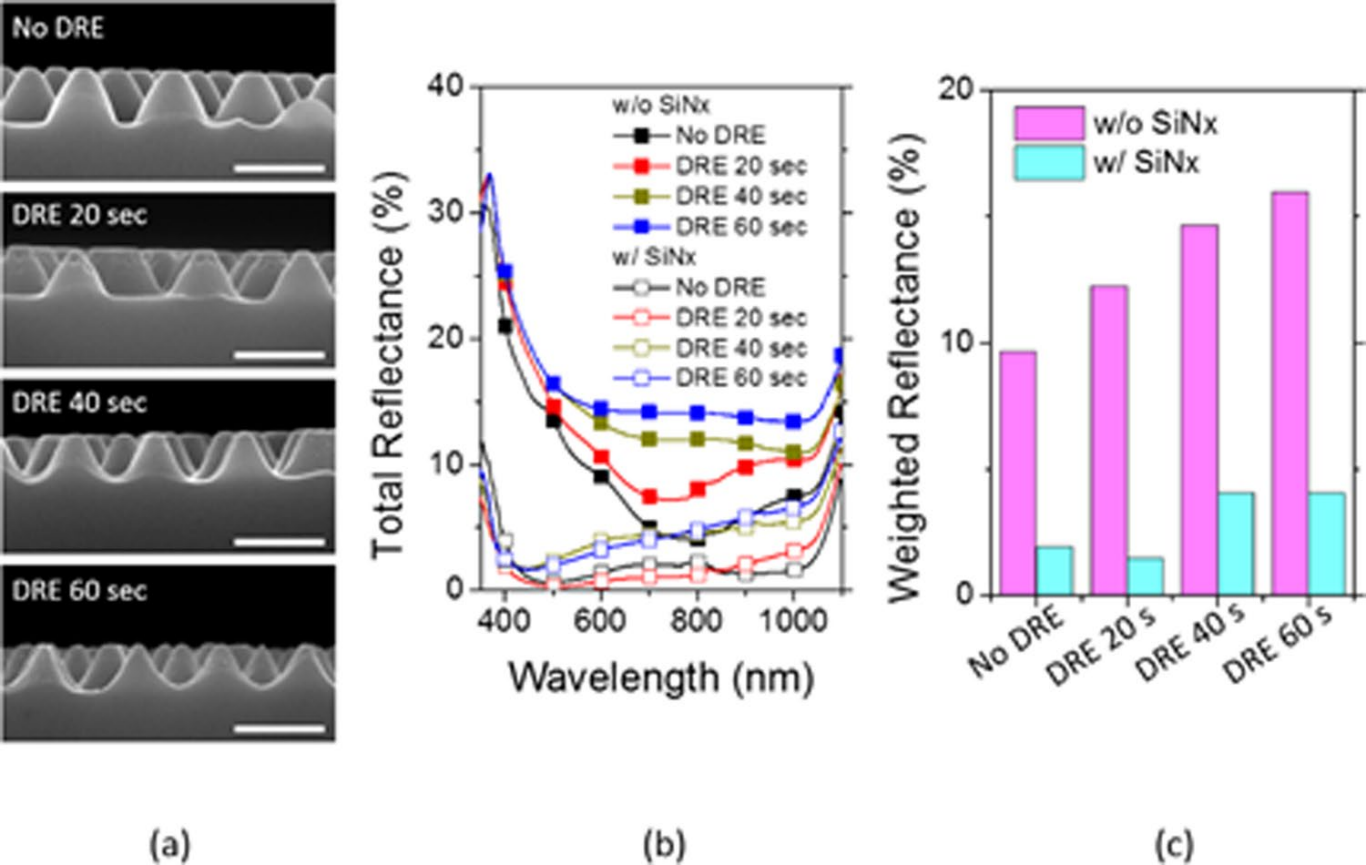
(**a**) Cross sectional SEM images of parabolic Si nanostructures with varying a DRE process time from 0 sec to 60 sec. (**b**) Reflectance curves and (**c**) weighted reflectances of parabolic Si nanostructures with or without SiNx. For comparison, reflectance values of a bare Si wafer is compared together. The scale bar is 500 nm for all the SEM images.

Fabrication of self-assembled silica bead monolayers in large are by spin coating is in general a challenging task^15^. The elaborate control of the environment of the spin coating processes such as humidity and solvent boiling temperature is demanded not to mention the spin coating parameters such as spinning speed and acceleration. Conventional solvents for spin coating are water and alcohol based ones^15, 28, 29^. In our previous study, we demonstrated that the use of the binary organic solvents of DMF (Dimethylformamide) and EG (Ethylene glycol) leads to a high quality self-assembled monolayers in large area. Following this approach, we spin-coated three different sized silica beads of 310 nm (P310), 520 nm (P520) and 960 nm (P960) in diameter on Si wafers treated with piranha. Optimal choices of silica bead concentrations depending on its size lead to formation of self-assembled monolayers of a high quality in large areas. Scanning electron microscopy (SEM) images in Fig. 1 show the silica beads of different sizes formed self-assembled monolayers of a high quality in a hexagonal lattice. The monolayer coverages of the silica bead monolayers were high over 90% for all the sizes of P310, P560, and P960, and the photograph images taken by a camera are shown in Fig. 1(d)-(f). The coverage was determined by calculating the area the silica bead monolayers occupy with image processing and normalizing to the perfectly close packed case. The different colors of the silica bead coated wafers in Fig. 1(d)-(f) arise from the optical interference depending on the size of the silica beads. Using the silica beads as an etch mask, we performed CF_4_/O_2_ reactive ion etch (RIE). The etch rate of the silica beads highly relies on the oxygen composition in the RIE gases^30–32^. Figure 2(a) indicates the higher fraction of oxygen leads to reduced silica etch rates, resulting in the increased etch selectivity, which is the etch rate ratio of Si to silica. The etch selectivity exhibit the highest of 0.63 at 10% O_2_ followed by a slight decrease with increasing the oxygen fraction. The maximum height of the Si nanostructures is determined by the selectivity because the silica bead is completely etched after a certain time. The CF_4_/O_2_ RIE process exhibits high anisotropy etch behavior. However, a spherical silica bead inherently has a different thickness along the radial direction; thus, the etched thickness of the Si wafers underneath the silica beads varies on the radial direction accordingly. By a combination of etch anisotropy and selectivity, parabola shaped silicon nanostructures can be formed as shown in a schematic of Fig. 3. The cross-section images of the Si nanostructures with different sizes of silica beads as etch masks show a parabola shape for all the three cases. Antireflection in broad spectral ranges by nanostructures on the Si wafers is attributed to the combined effects of strongly forward induced Mie scattering^33, 34^ and gradually increased effective index from air to the substrate^1, 35^. Suppression of reflectance in particular wavelength ranges is also caused by absence of high order reflective diffraction modes in the periodic nanostructures depending on the period^36^. The cut-off wavelength, which is the maximum wavelength where the first order of diffraction occurs, is proportional to the period ($$p)$$ of the nanostructures, and in the case of gratings in hexagonal arrays, the cut-off wavelength is $$\sqrt{3}/2\,\cdot \,p$$. In the wavelengths longer than the cut-off wavelength, the reflective diffraction is greatly suppressed. All the above effects contribute to broadband antireflections. The height, period and shape of the nanostructures are key factors to strong antireflection. Using a RCWA (Rigorous Coupled Wave Analysis) method, we simulated the weighted reflectances from the Si wafers with four different Si nanostructures: (1) curved cone, (2) cone, (3) parabola, and (4) ellipsoid. The weighted reflectance is obtained by calculation of the average reflectance in the wavelength range of 350 nm~1200 nm over a standard solar irradiation. The height and period of the nanostructures were varied in order to find optimal design guides. For all the cases, the coverage of the nanostructures was assumed to be 100%, and the fractional area of the bottom of the Si nanostructures on the wafers was kept at 81%, which is an experimentally achieved filling factor by our approach. In general, the nanostructures of higher aspect ratios lead to the lower reflectance irrespective of the nanostructure shapes. High aspect ratio Si nanostructures are optically strong light trapping nanostructures; however, the enlarged surface areas caused by the high aspect ratios are detrimental to charge carrier transport because of increased recombinations at the surface. Furthermore, the nanostructures are prone to be more heavily doped resulting in Auger recombination when they are used for emitter in the Si solar cells. In this regard, a shallower nanostructures with a reduced surface area would be more beneficial for solar cell applications^23, 37^. The height of the nanostructures is limited by the etch selectivity and the size of the silica beads in our approach. The period of the nanostructures is also determined by the silica bead size. Therefore, depending on the period, the maximum height is limited and denoted by the red dashed lines in Fig. 4. The white dashed lines indicate 1.0% of the weighted reflectance. The white dashed line in the case of the parabola shaped nanostructures lies well below the red dashed line in Fig. 4(c) in a wide period range (400 nm~800 nm), which means the parabola shaped nanostructures are more beneficial for antireflection even with shallower texturing compared with the other shapes. After introducing the parabolic Si nanostructures of three different periods of 310 nm, 520 nm and 960 nm on the Si wafers, the total reflectances were experimentally measured and shown in Fig. 5. The heights of the nanostructures for each nanostructure are 185 nm, 310 nm, 630 nm, respectively. As a reference, the reflectance of a bare silicon wafer without any texturing is compared together. With incorporation of the Si nanostructures, the reflectance decreases substantially compared with the reference as seen in Fig. 5(a). When the PECVD SiN_x_ layers are coated onto the Si nanostructures, the reflectances decrease further in broad spectral ranges especially from 400 nm to 1100 nm. The SiN_x_ layer reduces the reflectance by destructive interferences at the surface of the Si wafers. The thickness of the SiN_x_ was adjusted to have 70 nm, which is a quarter wave thickness particularly designed for a minimum reflectance at the wavelength of 550 nm. The weighted reflectance of the Si nanostructures of a 520 nm period (P520) exhibit the lowest value of 2.8%, which is superior to the most conventional pyramidal texturing in micro scale. The highest coverage and more ideal period for strong antireflection is considered to be the reason for the lowest weighted reflectance of the Si nanostructures among three sizes of the Si nanostructures. In contrast, the SiN_x_ coated planar wafer shows only a weighted reflectance of 10.2%. The Si nanostructures fabricated with P520 and P960 show higher weighed reflectances of 1.93% and 5.38%, respectively. The nanocone shaped Si nanostructures can be fabricated by using a SF_6_/O_2_ RIE process. We produced truncated nanocones of a 380 nm height with P520 silica spheres and SF_6_/O_2_ RIE etch and obtained slightly greater weighted reflectance values of 2.9% than the parabola Si nanostructures. The detailed results are described in the supplementary information. RIE processes in general induce damages at the surface of the wafers; thus, the damaged surface region needs to be removed by etching. We applied the isotropic acid etchant of HF:HNO_3_:CH_3_COOH (HNA) to remove the damage layer for very short time less than one minute^38^. We investigated the changes in the shape and size of the parabolic Si nanostructures with a damage removal process (DRE). We observed the SEM cross-section images of a 520 nm period with varying the DRE process time from 0 sec to 60 sec as shown in Fig. 6(a). Before the DRE process, the Si nanostructures show the parabola shaped Si nanostructures of a 310 nm height. The reflectances of the Si nanostructures increase consistently with increasing the DRE process time especially in long wavelength rages from 700 nm to 1100 nm as shown in Fig. 6(b). As the DRE process time increases, the height of the Si nanostructures decreases slightly, and only a 25 nm reduction in height is observed for a 60 sec DRE time. On the other hand, the shape of the nanostructures changes and becomes a more curved cone. The changes in the shape of the Si nanostructures with the DRE process is considered to play a main role in the increased reflectances as expected from the simulation results in Fig. 4. The weighted reflectances for the Si nano structures with varying the DRE process time were calculated and illustrated in Fig. 6(c). After deposition of a 70 nm SiNx layer, the nanostructures with a 20 sec DRE show a slight reduction in the weighted reflectance while the weighted reflectances after the DRE processes for 40 sec and 60 sec increase significantly.

### Device fabrication and characterization

**Figure 7:**
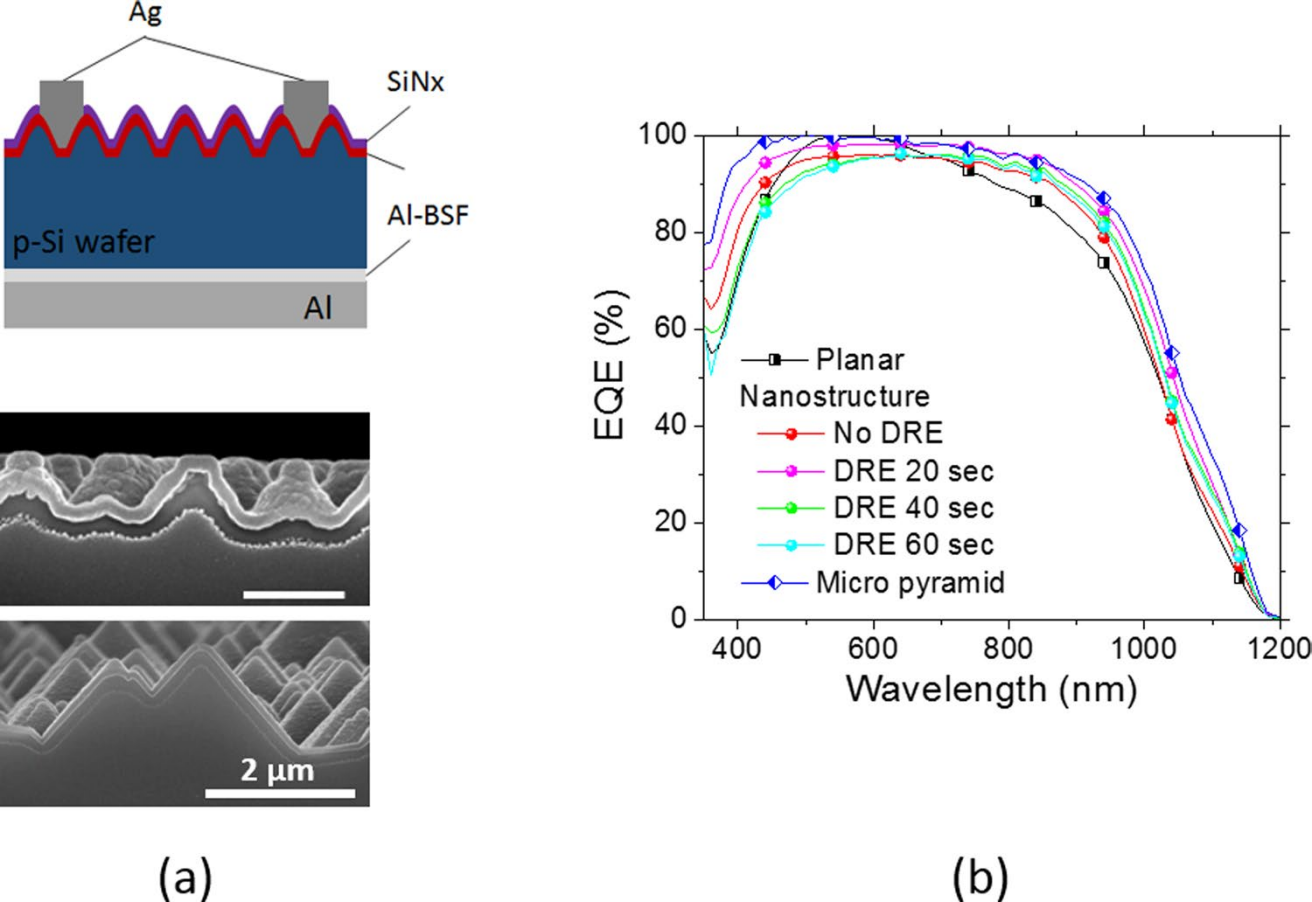
(**a**) Schematic of a solar cell with parabolic Si nanostructures of a standard device architecture (top). Cross sectional SEM images of the Si solar cells with nanostructures and micro pyramids (middle, bottom). A heavily doped emitter region beneath a SiNx overcoat layer of a 70 nm thickness was selectively etched by HNA solution. (**b**) EQE spectra of the solar cells textured with parabolic Si nanostructures by varying a DRE process time. For comparison, the EQE of a solar cell based on a planar wafer without texturing is plotted together. The scale bar in the SEM image of the parabolic Si nanostructures is 500 nm.

We fabricated Si solar cells of a standard structure with Al back surface field with introducing parabolic silicon nanostructures of a 520 nm period which provide the lowest weighted reflectance. The schematic of the device structure with parabolic Si nanostructures is illustrated in Fig. 7(a). Cell processes are described in the experimental section in detail. The solar cell based on the planar wafer without texturing exhibited a photocurrent of 35.5 mA/cm^2^ and an efficiency of 16.2%. With introducing the parabolic Si nanostructures, unexpectedly the photocurrent did not improve compared with the reference without texturing, and the efficiency dropped because of the decreased V_oc_. We performed a DRE process for a short time of 20 sec and observed increases in the photocurrent and the efficiency in turn. The efficiency was increased to 17.2%, which is attributed to an increased photocurrent of 37.7 mA/cm^2^. The external quantum efficiency (EQE) spectra reveal the increased photocurrent with the Si nanostructures are owing to the enhanced antireflection in broad spectral ranges as shown in Fig. 7(b). Without a DRE process, the EQE spectra show low values in the overall wavelength ranges. The RIE process induces surface damage as stated above. This damaged surface on the front side would decrease charge carrier collections in the emitter region. This leads to poor EQE values in short wavelength ranges below 700 nm. In addition, it is well known that carbon containing RIE gases leave hydrocarbon residues in the surface of the process wafers after an RIE process^39, 40^. This would decrease the EQE values in overall wavelength ranges. After a DRE process, the damaged layer and the hydrocarbon residues would be removed, resulting in the increased EQE values. We also observed the heavily doped region in the emitter by the cross-sectional SEM images in Fig. 7(a) ^21^. The selectively etched heavily doped region with a uniform thickness of ~100 nm is distinctly observed and follows the shape of the Si nanostructures. The uniform doping in the emitter region in the Si nanostructures is beneficial for high EQE values in the short wavelength regions. The Si nanostructures of high aspect ratios in emitter regions are prone to be more heavily doped by lateral dopant diffusion along the nanostructures resulting in poor EQE values in short wavelength regions. In order to suppress the lateral dopant diffusion, it is more desirable to use shallow nanostructures of a low aspect ratio. Our solar cell with the parabolic Si nanostructures after a DRE process exhibit 87.3% of EQE at the wavelength of 400 nm. This EQE value belongs to the highest group among the solar cells with texturing in nano scale and is comparable to that of the solar cells with micro pyramid texturing. Note that the prolonged DRE processes for 40 sec and 60 sec result in reduced EQE values particularly in the short wavelength rages of 350~600 nm. This might be caused by combined effects of the nanostructure shape changes into the curved cone and the lateral dopant diffusion^22^. For the purpose of comparative study, we also fabricated the solar cells based on conventionally textured wafers with microscale pyramids as a reference. The height of pyramids ranges from a few μm to 10 μm. The cross-sectional SEM image of the pyramid device is also shown in Fig. 7(a). The EQE values of the pyramid solar cell is slightly higher in a short wavelength range but comparable in an overall spectral range compared with those of the optimally designed nanostructure cells. The device parameters of the solar cells with planar and textured wafers depending on the DRE time are summarized in Table 1. Thanks to good antireflection with the parabolic Si nanostructures and the optimal DRE process of 20 sec, the highest solar cell efficiency of 17.2% comparable to the reference cell with the conventional pyramid texturing was achieved.

**Table 1:** Device parameters of solar cells with parabolic Si nanostructures by varying a DRE process time. For a control device, the solar cell based on a planar wafer is presented. The standard deviations are represented together with average values.

	No texturing	Nanodome w/o DRE	Nanodome DRE 20 sec	Nanodome DRE 40 sec	Nanodome DRE 60 sec	Micro pyramid
Efficiency (%)	16.2 ± 0.15	14.9 ± 0.24	17.2 ± 0.26	16.7 ± 0.21	16.5 ± 0.25	16.9 ± 0.27
J_sc_ (mA/cm^2^)	35.5 ± 0.21	35.3 ± 0.40	37.7 ± 0.42	36.6 ± 0.31	36.1 ± 0.62	38.9 ± 0.39
V_oc_ (V)	0.587 ± 0.001	0.564 ± 0.001	0.590 ± 0.003	0.584 ± 0.003	0.583 ± 0.001	0.589 ± 0.003
FF	0.778 ± 0.005	0.748 ± 0.004	0.775 ± 0.007	0.786 ± 0.003	0.785 ± 0.007	0.739 ± 0.010

In summary, we demonstrated the fabrication process of parabola shaped Si nanostructures by nanosphere lithography using self-assembly of the silica beads. The silica nanospheres were deposited onto the Si wafers by spin coating based on binary organic solvents of DMF and EG. Parabola shaped Si nanostructures, which were shown to be the most effective nanostructures for antireflection by the RCWA calculations, were fabricated by a single step of the CF_4_/O_2_ RIE process. By incorporation of the parabola shaped Si nanostructures, the reflectances were greatly decreased compared with the case of no texturing. The DRE process for a short time of 20 sec was enough to have a full recovery of the device parameters of *V*_*oc*_ and *FF* from the surface damages induced by the RIE process. The device efficiency was improved with introducing the parabola shaped nanostructures of the optimal size and period, which was 520 nm diameter and 310 nm height, mainly due to the improved photocurrent arising from enhanced antireflection.

## Methods

Crystalline Si wafers (1–5 Ω·cm, p-type, CZ) of a (100) crystal orientation of square pieces (30 mm × 30 mm) were washed in acetone, methanol, and ethanol with sonication for 15 min and were rinsed in deionized water for 10 min. In order to form a hydrophilic Si surface, they were subsequently cleaned in a piranha solution [H_2_SO_4_ (95%):H_2_O_2_ (30%) = 3:1] for 20 min followed by 10 min deionized water rinse. The spherical silica beads (Bang Laboratory) of 310, 520, and 960 nm in diameter were prepared for silica colloidal solutions. The silica beads of 310, 520, and 960 nm were dispersed in a binary solution [N,N-dimethyl formamide (DMF, anhydrous 99.8%, Sigma-Aldrich): Ethylene glycol (EG, anhydrous 99.8%, Sigma-Aldrich) = 9:1, v/v] at a silica bead concentration of 130 mg/ml, 185 mg/ml and 315 mg/mL, respectively. The silica bead solutions were sonicated for complete dispersion using a ultrasonic machine. The solution of 85 µL was dropped and spun onto the clean Si substrates using a spin coater (Spin Coater, ACE-200) at 2000 rpm for 375 s. The silica self-assembled monolayers (SAMs) in a hexagonal array were produced and used as dry etch masks to fabricate the Si nanostructures of a parabolic shape. The dry etching was performed in reactive-ion etching (RIE, Plasma Therm 790 Series) process for optimal control of the height and shape of the Si nanostructures. The composition of CF_4_/O_2_ mixed gas in RIE was adjusted from 0% to 15% in O_2_ ratio to investigate the selectivity of Si and silica beads. Well-fabricated silica SAMs of three different periods on silicon wafers were etched by CF_4_/O_2_ with 10% oxygen ratio. The SAMs of 310 nm, 520 nm and 960 nm in silica bead diameter were etched for 6 min 20 sec, 10 min 30 sec and 21 min, respectively until the silica beads were nearly completely removed. After etching process, the Si substrates were rinsed in 25 wt% HF solution for a few minutes to completely remove the residual silica beads. The total reflectance of the Si substrates was measured in the wavelength range from 350 nm to 1100 nm by UV/Vis-NIR spectroscopy (Cary 5000, Agilent) with a calibrated the integrating sphere as a reference. The total reflectance of the parabolic Si nanostructures in the wavelength ranges of 350 nm~1200 nm was simulated by varying the shape, height and period of the nanostructures using the RCWA technique to find the design guidelines for broadband antireflection. The commercial simulation package (RSoft Diffractmod) was used for the calculations in three dimensions. For accurate calculations, the spatial harmonics were set to be 5 and the index resolution in the vertical direction was adjusted to be 1/20 of the Si nanostructure height. The arrays of Si nanostructures were assumed to be hexagonal with a fixed 81% of filling factor which was adjusted by calculating the diameter of the bottom of Si nanostructures in proportion to each period. The Si substrate coated with a 70 nm thickness of a conformal overcoat layer was set to be semi-infinite for the total reflectance calculations. The refractive index of the overcoat layer was set for 1.9, which was approximately that of SiN_x_. The various shapes of the Si nanostructures such as curved cone, cone, parabola and ellipsoid were tested for the calculations. The silicon wafers with nanostructure texturing were cleaned by organic cleaning process and RCA1 cleaning. The Si substrates were rinsed in 25 wt% HF solution for a few minutes to remove the native oxide, followed by deionized water rinse for several minutes. The surface damage of the nanostructures induced by the RIE process was removed by wet etching using HNA solution of HF: HNO_3_: CH_3_COOH = 1: 75: 50 for 0 sec, 20 sec, 40 sec and 60 sec. The shape of the nanostructures were observed by FE-SEM (Nova-SEM), and total reflectance of the Si wafers were measured by UV/Vis-NIR spectroscopy. After RCA1 and RCA2 cleaning, a 2 μm thickness of Al was evaporated onto the back side of Si wafers at a deposition rate of ~15 nm/min using an electron-beam evaporation system. After organic cleaning process, a phosphorus spin on diffusant (SOD P507, Filmtronics) was spun onto the polished side of silicon wafers using a spin coater at 3000 rpm for 15 sec, and then wafers were put in the oven at 130 °C for 10 min. During heat treatment at 900 °C for 57 sec using a rapid thermal annealing system (RTA), n^+^ emitter was formed with a sheet resistance of 85~110 Ω/◽ on the front side, and p^+^ Al back surface(Al-BSF) was simultaneously formed on the rear side of the Si wafers. Phosphor-silicate glass (PSG) on the Si wafer surface was removed by HF solution, followed by RCA1 and organic cleaning. Ti/Ag front electrode patterns were formed by photolithography and a lift-off process. The SiN_x_ layer of which refractive index is approximately 1.9 at the wavelength of 550 nm was evaporated at a 70 nm thickness for passivation and antireflection using a plasma enhanced chemical vapor deposition system at 400 °C (Plasmalab 800 Plus, Oxford). Four neighboring cells of 10 mm × 10 mm were isolated by a SF_6_ RIE etch process. Al of a 2 μm thickness was evaporated on the back side of the cells for electrical contact at the bottom side. Finally, a forming gas anneal (FGA) process was performed at 350 °C for 40 min in a tube furnace. An alkaline solution of 45 wt% KOH was used for microscale pyramid texturization. The same cell fabrication processes were applied for the reference solar cell based on the wafers textured with microscale pyramids. All the cells were tested at room temperature under a solar simulator (Oriel LCS-100) with AM 1.5 G solar irradiation at a light intensity of 100 mW/cm^2^.

## Electronic supplementary material

Supplementary information
