# Supplementary material for: Fabrication of parabolic Si nanostructures by nanosphere lithography and its application for solar cells
Source: Sci Rep. 2017 Aug 4;7:7336. doi: 10.1038/s41598-017-07463-7 (PMC5544770; doi:10.1038/s41598-017-07463-7)
Supplement: Supplementary file 1 — Supplementary information [file 41598_2017_7463_MOESM1_ESM.doc]

**Supplementary information for**

**Fabrication of parabolic Si nanostructures by nanosphere lithography and its application for solar cells**

**See-Eun Cheon^1,2^, Hyeon-seung Lee^1,2^ , Jihye Choi^1^, Ah Reum Jeong^4^, Taek Sung Lee^1^, Doo Seok Jeong^1^, Kyeong-Seok Lee^1^, Wook-Seong Lee^1^, Won Mok Kim^1^, Heon Lee^2^, Inho Kim^1*^**

*^1^ Electronic Materials Research Center, Korea Institute of Science and Technology, Seoul 02792, Republic of Korea*

*^2^ Department of Materials Science and Engineering, Korea University, Seoul 02841, Republic of Korea*

*^3^ Department of Materials Science and Engineering, Yonsei University, Seoul 03722, Republic of Korea*

*^4^Division of Nano & Information Technology, Korea University of Science and Technology, Seoul 02792, Republic of Korea*

1. Fabrication of truncated Si nanocones and optical properties


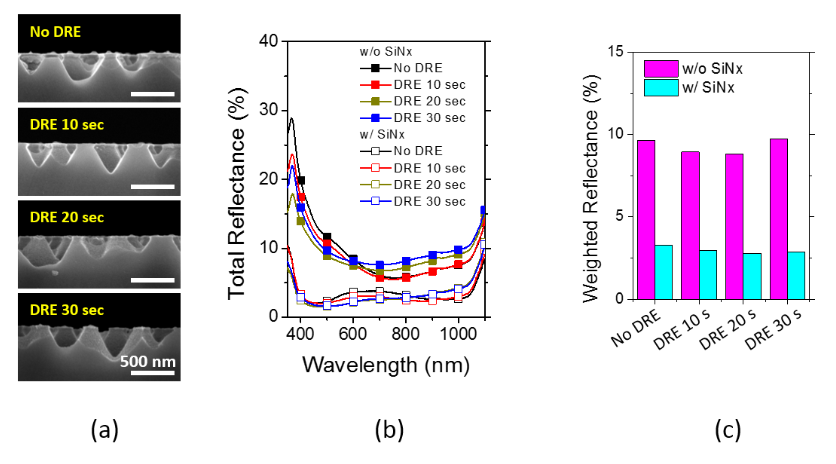


Figure S.1. (a) Cross-sectional SEM images of truncated nanocones produced by P520 silica nanospheres and SF_6_/O_2_ (O_2_ 40%) RIE processes. (b) Total reflectance spectrum curves of truncated nanocones with varying a DRE process time for the cases with and without SiNx of a 70 nm thickness. (c) Weighted average reflectance values for different nanocones with varying a DRE process time.
